# Development of a microRNA-Based age estimation model using whole-blood microRNA expression profiling

**DOI:** 10.1016/j.ncrna.2025.03.003

**Published:** 2025-03-03

**Authors:** Yanfang Lu, Anqi Chen, Mengxiao Liao, Ruiyang Tao, Shubo Wen, Suhua Zhang, Chengtao Li

**Affiliations:** a School of Forensic Medicine, Shanxi Medical University, Taiyuan, Shanxi, 030009, China; b Institute of Forensic Science, Fudan University, Shanghai, 200032, China; c Shanghai Key Laboratory of Forensic Medicine, Shanghai Forensic Service Platform, Academy of Forensic Science, Shanghai, 200063, China

**Keywords:** Age estimation, MicroRNA, Machine learning

## Abstract

Age estimation is a critical aspect of human identification. Traditional methods, reliant on morphological examinations, are often suitable for living subjects. However, there are relatively few studies on age estimation based on biological samples, such as blood. Recent advancements have concentrated on DNA methylation for forensic age prediction. However, to explore further possibilities, this study investigated microRNAs (miRNAs) as alternative molecular markers for age estimation. Peripheral blood samples from 127 healthy individuals were analyzed for miRNA expression using small RNA sequencing. Lasso regression selected 103 candidate miRNAs, and Shapley additive explanations (SHAP) analysis identified 38 key miRNAs significant for age prediction. Five machine learning models were developed, with the elastic net model achieving the best performance (MAE of 4.08 years) on the testing set, surpassing current miRNA age estimation results. Additionally, we observed significant changes in the expression levels of miRNAs in healthy individuals aged 48–52 years. This study demonstrated the potential of blood miRNA biomarkers in age prediction and provides a set of miRNA markers for developing more accurate age prediction methods.

## 1 Introduction

Chronological age is one of the key characteristics of individual identity. Accurately estimating the age of an unknown individual not only helps confirm the identity of the person, but also helps narrow the scope of identification in judicial cases. Therefore, developing more accurate and reliable age estimation methods has always been an important research direction. Traditional age estimation primarily relies on morphological measures such as analyzing the characteristics of the skeleton and teeth [1,2]. However, these methods are often only applicable to live studies, and the samples required are difficult to obtain at crime scenes.

In recent years, with a deeper understanding of the aging mechanism, molecular-level age markers have received increasing attention. DNA methylation is currently a research hotspot in the field of age prediction. Hannum et al. [3], Horvath et al. [4] and Weidner et al. [5] employed DNA methylation data to establish models for evaluating aging, known as "epigenetic clocks", thereby paving the way for age estimation. Subsequently, a series of forensic studies have reported the establishment of DNA methylation age epigenetic clocks, with prediction accuracy reaching around 3–5 years [6,7]. Although DNA methylation analysis has shown great potential for application in forensics, to explore more possibilities in age prediction, researchers began to turn to RNA.

RNA is widely involved in the regulation of life activities, and its expression pattern is closely related to individual development and aging. Transcriptome analysis provides a new approach for age prediction. Bauer [8] systematically discussed the application prospects of RNA molecules in the field of forensics in 2007, highlighting the potential of RNA in forensic medicine. In 2015, Peters et al. [9] based on peripheral blood transcriptome expression profile analysis, first identified 1497 age-related differentially expressed genes, and established a transcriptome-based age prediction model, confirming the potential of transcriptome molecules for age prediction. Subsequently, some studies have attempted to construct age prediction models using transcript markers such as mRNA and microRNA (miRNA) [10,11]. Among them, miRNA has received much attention due to its advantages such as short fragment length, high stability, and good detection sensitivity [12]. It is well known that miRNAs regulate various aspects of cellular activities, including differentiation and development, metabolism, proliferation, apoptosis, viral infection, and tumorigenesis [13,14]. Several studies have shown that many miRNAs are also involved in the regulation of aging-related pathways, and their expression levels can serve as potential age markers [[15], [16], [17], [18], [19], [20], [21], [22], [23]].

Although the role of miRNAs in human aging has attracted widespread attention [24,25], research on the use of miRNAs for age estimation is still relatively limited. In 2020, Fang et al. [26] used massive parallel sequencing to analyze the miRNA expression profiles of peripheral blood samples from 100 Chinese Han adults (20–69 years). Through Pearson correlation analysis, they identified six miRNA biomarkers that were significantly associated with aging. To further validate the predictive performance of these miRNA biomarkers, the researchers used RT-qPCR to detect the peripheral blood samples of another 120 subjects and established an AdaBoost age prediction model based on these six miRNAs, achieving a mean absolute error (MAE) of 6.461 years. Although this study confirmed the potential of using miRNAs for age prediction, further exploration of samples with a larger age range, more accurate modeling methods, and validation of their application value in forensic practice are still needed.

In this study, we conducted a large-scale study on the miRNA expression profiles of the Chinese Han adult population. We collected 127 peripheral blood samples from the Chinese Han population aged 18–81 years, essentially encompassing the spectrum of adulthood. After detecting the miRNA expression profiles by small RNA sequencing, a subset of 38 miRNAs was selected through lasso regression and Shapley additive explanations (SHAP) analysis. Five machine models were compared for accuracy in predicting age, with the elastic net and support vector machine found to be the most accurate. The study validated the potential of miRNAs as age estimation markers, thereby offering a novel approach for subsequent age-related miRNA screening and model development.

## 2 Materials and methods

### 2.1 Sample collection and RNA isolation

A total of 127 peripheral blood samples (63 men and 64 women) were collected from a healthy Chinese Han population, ranging in age from 18 to 81 years. Participants were recruited at one-year intervals, with one male and one female each year. The samples were stored at −20 °C until RNA extraction and analysis. Informed consent was obtained from all individual participants included in the study before sample collection. The study was approved by the Ethics Committee of the Academy of Forensic Science. Ministry of Justice, P.R. China (NO.2022–11).

Total RNA was isolated from the peripheral blood samples using the TRIzol reagent (Invitrogen, USA) according to the manufacturer's protocol. RNA concentration was measured using the Qubit RNA HS Assay Kit and Qubit 4.0 Fluorometer (ThermoFisher Scientific, USA). RNA purity was assessed with a NanoDrop 2000 spectrophotometer (ThermoFisher Scientific, USA), ensuing a 260 nm/280 nm absorbance ratio between 1.8 and 2.1 for all samples. RNA integrity was evaluated using an Agilent 2100 Bioanalyzer and the Agilent RNA 6000 Nano Kit (Agilent, US), with all samples showing an RNA Integrity Number (RIN) greater than 7. The extracted RNA was aliquoted into RNase-free microcentrifuge tubes and stored at −80 °C to ensure RNA stability until the subsequent sequencing analysis.

### 2.2 Sequencing and data analysis

Small RNA sequencing libraries were prepared using 100 ng of total RNA from each sample. To verify the reproducibility and consistency of the miRNA sequencing results, 20 libraries were selected for replicate sequencing. The libraries were generated using the QIAseq miRNA Library Kit (Qiagen, Germany) according to the manufacturer's instructions. Briefly, the 3′ and 5′ adapters were ligated to the RNA, followed by reverse transcription and PCR amplification. The amplified libraries were purified and size-selected using QIAseq miRNA NGS beads (Qiagen, Germany). The quality and quantity of the libraries were assessed using the Agilent High Sensitivity DNA Kit on the Agilent 2100 Bioanalyzer (Agilent, USA).

The libraries were sequenced on the Illumina Hiseq 2500 platform (Illumina, USA) with 50 single-end reads. Raw sequencing data was first evaluated for quality using FastQC. Adapter sequences and low-quality reads were removed using Trimmomatic. Clean reads were then aligned to the human reference genome (GRCh38) using the Burrows-Wheeler Aligner (BWA). MiRNA identification and expression quantification were performed using miRDeep2, with annotation and naming based on the miRBase database.

We normalized the miRNA expression data across all samples to eliminate differences in expression magnitudes between different miRNAs, improving the convergence speed and stability of the model [27]. The formula for calculating the CPM (counts per million) value of each miRNA in each sample is as follows:

$$CPM=\frac{A\;miRNA\;read\;counts}{Total\;read\;counts}\times 10^{6}$$

### 2.3 Identification of differentially expressed miRNAs

To thoroughly investigate the effect of age on miRNA expression, samples were divided into 13 groups, each representing a 5-year age range. We adopted two different grouping strategies using the edgeR package to screen for differentially expressed miRNAs. The first method involved conducting differential expression analysis between each age group and its two adjacent two age groups. The second method involved comparing each group to the first group (18–22 years) for differential analysis. The Benjamini-Hochberg method was applied to correct the original p-values for multiple hypothesis testing. MiRNAs were considered differentially expressed if the adjusted p-value <0.05 and the |log2FC| > 1. All statistical analyses in this study were conducted using R software.

### 2.4 Functional analysis using GO and KEGG pathway

Based on the differential expression analysis, we selected both upregulated and downregulated miRNAs for detailed functional studies using DAVID tool. According to the classification of gene functions by Gene Ontology (GO), there are three main categories: biological process, molecular function, and cellular component. These analyses help elucidate the potential roles of miRNAs in cellular structures, biological processes, and molecular functions, providing important clues for further research. Additionally, we conducted pathway analysis using Kyoto Encyclopedia of Genes and Genomes (KEGG) to complement GO analysis, offering a more comprehensive understanding of the regulatory functions of these miRNAs at the cellular biology level.

### 2.5 Selection of age-related miRNAs

To screen for additional age-associated miRNA, lasso regression is utilized to select features. Lasso regression is a widely applied penalized linear regression model ideal for high-dimensional data, facilitating dimensionality reduction and feature selection. This method is particularly suitable for our study, where the number of variables far exceeds the number of samples, as it can effectively reduce model complexity and prevent overfitting. This step used the CPM values of miRNA expression. We used 10-fold cross-validation to optimize the lasso regression parameters, ensuring the model avoids overfitting while retaining a sufficient number of age-related miRNA features. Non-zero coefficient variables were selected as candidate age-related miRNAs that significantly influence age prediction.

To further determine age-related miRNAs, we employed SHAP analysis to decompose the candidates identified by lasso regression. By calculating the SHAP values for each candidate to explain their contribution to age prediction, we selected those with SHAP values above the average of all features as significant. This process allowed us to identify key miRNAs for predicting age, providing more reliable and robust candidate biomarkers for subsequent analysis. The miRNAs associated with age prediction were subjected to linear regression analysis to assess the linear relationship between miRNA expression levels and age. Statistical significance was determined with p-value < 0.05, and the strength and direction of a relationship was represented by the correlation coefficient (r).

### 2.6 Establishment of machine learning models

Using the R statistical software, we constructed five different machine learning models-random forest (RF), support vector machine (SVM), XGBoost, elastic net and neural network-to evaluate the performance of candidate miRNAs in the age prediction task. Machine learning has wide applications in the medical field, particularly for modeling and predicting complex biological processes such as the aging [28].

We used a stratified sampling method to randomly split all the sample data into a training set and a testing set at an 8:2 ratio, ensuring consistency of age distribution between the sets. For each model, we systematically searched a pre-defined set of hyperparameter combinations using grid search and selected the optimal hyperparameter combination based on 10-fold cross-validation performance metrics on training set and testing set. To comprehensively evaluate the performance of the different models, we adopted the mean absolute error (MAE) to measure the average absolute difference between the actual age and the predicted age, reflecting the accuracy of the model predictions.

## 3 Results

### 3.1 Overview of sequencing results

Data quality is important for ensuring the reliability of the subsequent analysis. Our sequencing results show that the small RNA length distribution in all samples is primarily concentrated between 21 and 24 bp, with 22 bp small RNAs being the most abundant, indicating the presence of a substantial number of miRNAs. In this study, we identified 1422 miRNAs through small RNA sequencing from all peripheral blood samples. The average total reads for all samples was 32.74 million, with an average of over 27.21 million miRNA reads, accounting for 83.11 % of total reads. Furthermore, the results of repeated sequencing for 20 samples demonstrated no significantly differentially expressed miRNAs between two sequencing runs. The repeated sequencing confirmed the robustness and reproducibility of our miRNA expression data.

### 3.2 Analysis of differentially expressed miRNAs

**Fig. 1:**
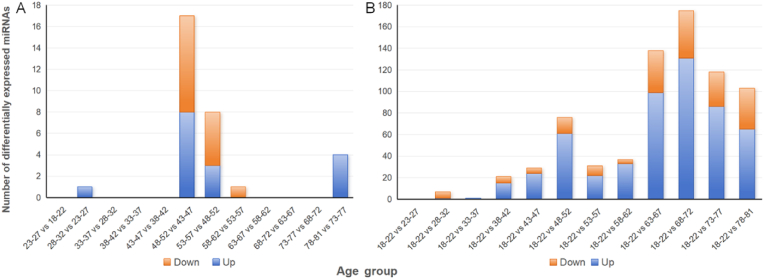
Grouped the samples by age in 5-year intervals to identify differentially expressed miRNAs. (A) The number of miRNAs between each age group and its adjacent groups. (B) The number of miRNAs between the first group and each of the other groups.

**Fig. 2:**
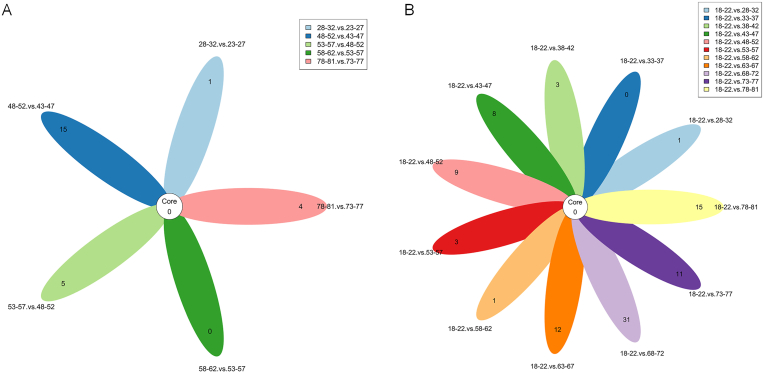
Flower plot of differentially expressed miRNAs identified in different groups. The number at the center of the flower plot indicate the miRNAs common to all groups, while the numbers on the petals indicate the miRNAs unique to each group. (A) Between adjacent groups. (B) Between each group and the first group.

To identify age-associated miRNA expression changes, we grouped the samples into 5-year age intervals and performed differential expression analysis between adjacent groups as well as between the first group (18–22 years) and each of the other groups (Fig. 1). Among the adjacent age groups, the 48–52 age group exhibited the most significant number of differentially expressed miRNAs. Specifically, 17 miRNAs were differentially expressed between the 48–52 and 43–47 age groups, and an additional 8 miRNAs were identified in the comparison between the 48–52 and 53–57 age groups. When examining the 10-year age intervals, the 48–57 age group showed the most significant miRNA expression changes. More importantly, from the age of 48–52 age group, the number of differentially expressed miRNAs significantly increased compared with the first group. However, in the analysis results, we did not find any overlap in the differentially expressed miRNAs identified in each group (Fig. 2).

### 3.3 Functional enrichment analysis

To further explore the differential expression of miRNAs in the 48–52 age group, we conducted GO analysis and KEGG pathway analysis. The GO analysis revealed that miRNAs differentially expressed across each 5-year age group were significantly enriched in biological processes related to metabolic process and biosynthetic process. At the molecular function level, these miRNAs were predominantly enriched in RNA binding and nucleic acid binding. Cellular composition analysis showed that these miRNAs were mainly located in intracellular and nucleus.

**Fig. 3:**
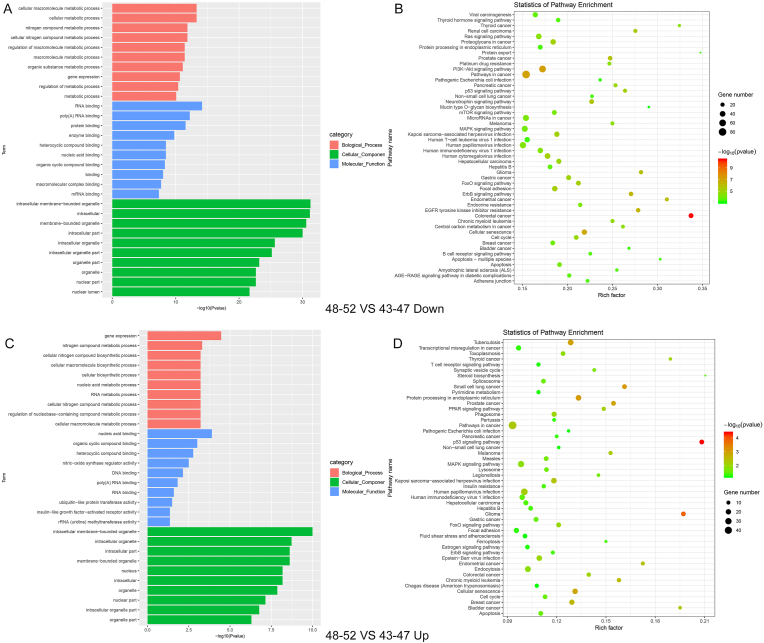
The GO and KEGG analysis results of miRNAs identified between the 48–52 and 43–47 age groups. (A) The GO analysis results for 48–52 vs 43–47 down-regulated miRNAs. (B) The KEGG analysis results for 48–52 vs 43–47 down-regulated miRNAs. (C) The GO analysis results for 48–52 vs 43–47 up-regulated miRNAs. (D) The KEGG analysis results for 48-52 vs 43–47 up-regulated miRNAs.

**Fig. 4:**
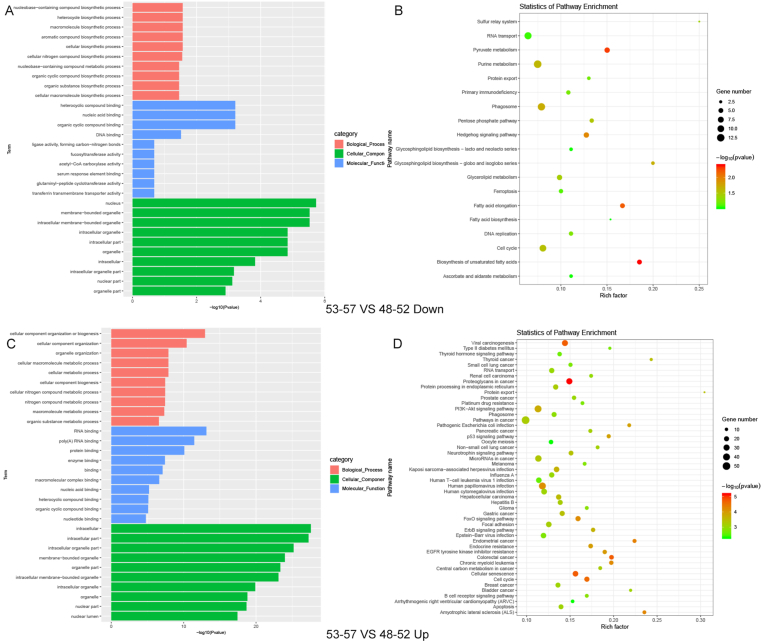
The GO and KEGG analysis results of miRNAs identified between the 57-57 and 48–52 age groups. (A) The GO analysis results for 53–57 vs 48–52 down-regulated miRNAs. (B) The KEGG analysis results for 53–57 vs 48–52 down-regulated miRNAs. (C) The GO analysis results for 53–57 vs 48–52 up-regulated miRNAs. (D) The KEGG analysis results for 53–57 vs 48–52 up-regulated miRNAs.

KEGG pathway analysis revealed an enrichment of these miRNAs in biosynthesis, metabolism and cancer-related signaling pathways, such as the colorectal cancer and p53 signaling pathway. Fig. 3, Fig. 4 showed the KEGG and GO analysis results of miRNAs identified between the 48–52 and 43–47 age groups, as well as between the 48–52 and 53–57 age groups, respectively.

### 3.4 Selection of age-related miRNAs and development of age prediction models

To obtain more age-related miRNAs, we identified 103 age-related markers by lasso regression method from the initial set of 1422 miRNAs. Subsequently, we utilized five machine learning algorithms to construct age prediction models based on these 103 miRNAs, aiming to comprehensively evaluate their potential for age prediction. Among all evaluated models, the elastic net model performed the best. On the training set, the elastic net model achieved an MAE of 2.62 years and an R^2^ of 0.964, indicating excellent model fit. More importantly, on the independent testing set, the model maintained an MAE of 3.91 years and an R^2^ of 0.935, demonstrating its outstanding generalization ability and robustness.

The SVM model also performed well, with an MAE of 4.03 years and an R^2^ of 0.927 on the testing set. Although slightly outperformed by the elastic net model, SVM remains a powerful and widely-used machine learning algorithm. In contrast, XGBoost and RF exhibited relatively poorer performance, with MAEs of 6.30 years and 8.70 years on the testing set, respectively. The neural network model performed reasonably well on the training set, with an MAE of 3.49 years and an R^2^ of 0.926. However, its performance deteriorated significantly on the testing set, with an MAE of 7.63 years and an R^2^ of 0.726 (Table 1).

**Table 1:** Results of five machine learning models constructed using 103 age-related microRNAs.

Model	Training set		Testing set	
	MAE (years)	R^2^	MAE (years)	R^2^
RF	6.11	0.825	8.70	0.748
SVM	3.01	0.939	4.03	0.927
XGBoost	4.20	0.911	6.30	0.845
Elastic Net	2.62	0.964	3.91	0.935
Neural Network	3.49	0.926	7.63	0.726

**Fig. 5:**
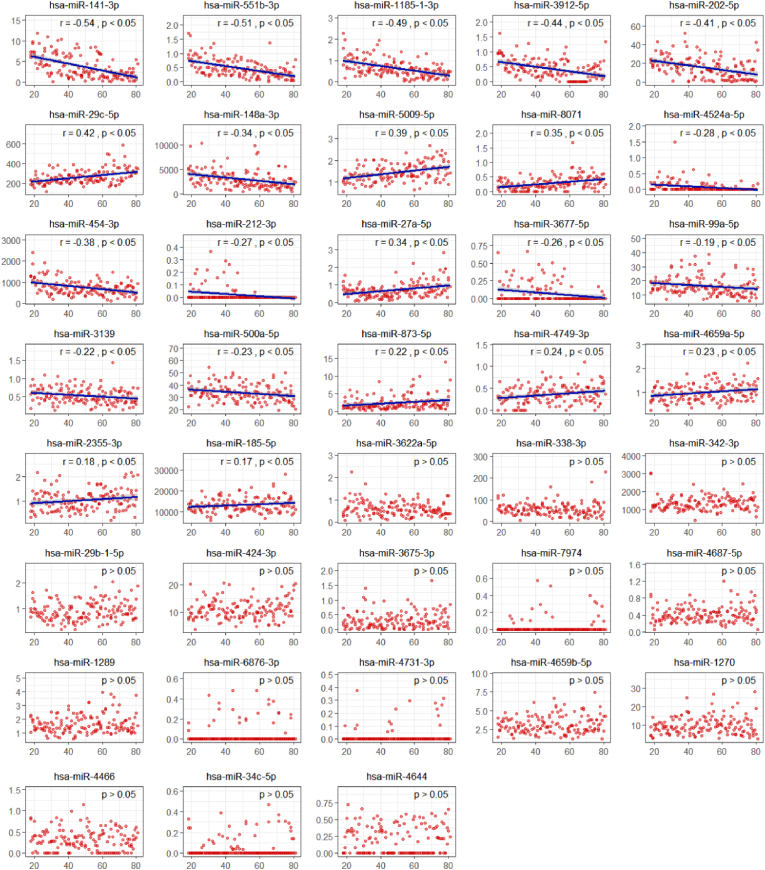
Scatter plot illustrating the linear relationships of 38 age-associated miRNAs.

To further optimize model performance, we employed the SHAP method to elucidate the optimal models (elastic net and SVM) utilizing the 103 age-associated miRNAs. SHAP-based feature selection identified 51 significant miRNA features for the SVM model and 44 significant miRNA features for the elastic net model. To obtain a stable and highly correlated set of miRNA features, we took the intersection of the miRNAs selected by the two models, ultimately identifying 38 commonly enriched significant miRNAs. These 38 miRNAs will be used as feature inputs for subsequent model validation, aiming to improving the predictive performance and biological relevance of the models. The linear regression analysis indicated that among the 38 selected miRNAs, 22 exhibited a linear relationship between their expression levels and age (p < 0.05). Among these, 9 miRNAs showed a positive correlation (correlation coefficients ranging from −0.23 to −0.54), while 13 miRNAs demonstrated a negative correlation (correlation coefficients ranging from 0.17 to 0.42). Fig. 5 displays the scatter plot of 38 age-related miRNAs, highlighting their linear correlation.

**Fig. 6:**
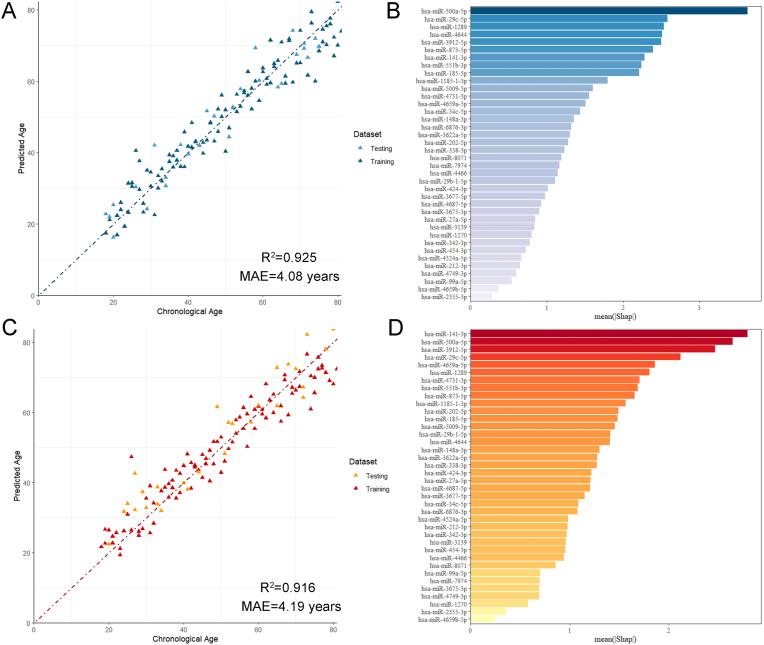
Performance of Elastic Net and SVM models using 38 age-related microRNAs and the mean absolute SHAP values distribution of miRNAs. (A) Chronological age against predicted age for elastic net model. (B) The mean SHAP values of the elastic net model for 38 miRNAs. (C) Chronological age against predicted age for SVM model. (D) The mean SHAP values of the SVM model for 38 miRNAs.

Based on the SHAP analysis, we screened a subset of 38 significantly relevant miRNA markers as the optimized feature set to evaluate the performance of five machine learning algorithms, adopting the same modeling workflow as before. The results showed that the elastic net model achieved an MAE of 3.89 years and an R^2^ of 0.931 on the training set, maintaining similar performance on the testing set (MAE = 4.08 years, R^2^ = 0.925) (Fig. 6A, Supplementary Table 1). The SVM model performed slightly worse than the elastic net, with an MAE of 4.19 years and an R^2^ of 0.916 (Fig. 6C–Supplementary Table 2). In comparison, the remaining three models exhibited poorer performance on the age prediction task, with testing set MAEs ranging from 7.12 to 9.60 years (Table 2). We conducted SHAP analysis on the 38 miRNAs in the elastic net model and SVM model. Based on the mean absolute SHAP values, the most important miRNA in the elastic net model was miR-500a-5p (Fig. 6B), while the most important miRNA in the SVM model was miR-141-3p (Fig. 6D). Detailed mean absolute SHAP values for the 38 miRNAs in both models are provided in Supplementary Table 3.

**Table 2:** Results of five machine learning models constructed using 38 age-related microRNAs.

Model	Training set		Testing set	
	MAE (years)	R^2^	MAE (years)	R^2^
RF	6.53	0.901	9.60	0.742
SVM	4.07	0.927	4.19	0.916
XGBoost	5.62	0.833	7.12	0.814
Elastic Net	3.89	0.931	4.08	0.925
Neural Network	3.89	0.928	7.58	0.734

Furthermore, to observe the impact of sex on prediction, we conducted separate modeling analyses on male and female sample subsets. The experimental results showed that the elastic net and SVM models generally performed better on the male samples than on the female samples. The elastic net model achieved the best performance in both sex subsets, with an MAE of 4.14 years for male samples and 6.61 years for female samples (Table 3).

**Table 3:** Elastic Net model performance in total samples, male and female groups.

Elastic Net	Training set		Testing set	
	MAE (years)	R^2^	MAE (years)	R^2^
Female (n = 63)	4.28	0.901	6.61	0.894
Male (n = 64)	3.01	0.972	4.14	0.940
All (n = 127)	3.89	0.931	4.08	0.925

## 4 Discussion

Age inference for unknown individuals is important in human identification. Previous research has demonstrated that epigenetic age prediction is currently the most accurate and widely studied approach [29,30]. To explore more possibilities, RNA has garnered increasing interest among researchers. Studies have shown that forensic samples can yield sufficient quality and quantity of RNA [31], and that RNA and DNA can be co-extracted, enhancing the detectability of challenging samples [[32], [33], [34], [35]]. Among the various types of RNA, miRNA is recognized for its enhanced resistance to degradation, attributed to its small size and ability to bind to proteins, which provides a significant advantage in forensic science. miRNA can be stably stored at room temperature for at least 48 h and retains stability even after multiple freeze-thaw cycles [36]. Grabmüller et al. [37] successfully extracted useable miRNA from the preserved PVAL gloves that had been stored for 20 years. In forensic applications, miRNA in blood samples demonstrates considerable stability under varying environmental conditions. Although exposure to high temperatures (95 °C) and ultraviolet radiation for 7 days can compromise miRNA stability, the degradation rate remains relatively slow, allowing for successful detection [38,39]. At lower temperatures, particularly at −80 °C, miRNA in blood exhibits enhanced stability [12,40], remaining unchanged even after 4 years of storage [41]. Significant correlation has been observed between miRNA expression levels and individual age. Fang et al. [26] first reported an age prediction model using miRNA from the peripheral blood of the Chinese Han population, achieving a MAE of 6.461 years. This finding provided preliminary evidence of the potential application of miRNA in forensic science. However, compared to "epigenetic clock" based on DNA methylation, which has a MAE of approximately 3–5 years, the accuracy of miRNA prediction models still requires further improvement. Numerous studies have demonstrated significant differences in miRNA expression across various populations. For example, Wu et al. [42] employed microarray technology to compare the miRNA expression profiles of normal skin tissues between Uyghur and Han individuals. Their analysis showed that among 3100 microarray probes, 247 miRNAs exhibited significant expression differences between these two groups. Similarly, Huang et al. [43] utilized microarray method to investigate the miRNA expression profiles of normal HapMap lymphoblastoid cell lines derived from individuals of Northern and Western European descent in Utah, as well as Yoruba individuals from Ibadan, Nigeria. Their findings indicated that 16 % of the miRNAs analyzed showed significant differences between these populations. Furthermore, many researchers have studied the significant differences in miRNA expression between different populations, even within the same disease context (including breast cancer [44,45], thyroid cancer [46], and ovarian cancer [47], among others [48,49]). This observation suggests that the peripheral blood miRNA expression profiles of healthy individuals from different populations may also exhibit significant differences. Consequently, we propose that the establishment of age-based predictive models tailored to specific populations is of paramount importance.

In this study, we utilized small RNA sequencing to detect and analyze 127 peripheral blood samples from Chinese Han individuals. The average sequencing total reads across all samples was 32.74 million, with an average of 27.21 million miRNA reads, accounting for 83.11 % of the total reads. According to industry standards and research, a sequencing depth of 1–10 million reads per sample is sufficient for downstream analysis [50]. Our sequencing results far exceeded this standard, providing a higher sequencing depth that is beneficial for the detection of low-abundance miRNA expression differences and more accurate quantification of miRNA expression levels [51]. Generally, miRNA accounts for 70–90 % of the total reads; in our study, the proportion of 83.11 % falls within this range, indicating the high reliability of our sequencing data. Furthermore, we analyzed 20 replicate sequencing samples and found that all miRNAs did not show significant differences in expression between the two replicates. These results suggested that our sequencing data is of high-quality, with good reproducibility and consistency, laying a solid foundation for subsequent analysis.

To identify age-related miRNAs, we first conducted a standard differential expression analysis. However, we noticed that not every group exhibited common miRNAs. This suggested that we may need to expand the scope of our study to cover a wider age range in order to find more specific age-related miRNAs. But we still found that the miRNA differential expression was most significant in the 48–52 age group. This result is consistent with the study by Maider et al. [52], which discovered significant changes in the expression levels of many small non-coding RNAs, including miRNA, in healthy individuals aged 47–54. To further investigate the reasons for the differential expression of miRNAs in the 48–52 age group, we conducted enrichment analysis. The result showed that these miRNAs are primarily enriched in metabolic processes and synthetic pathways. As the current study, miRNA mainly regulates the expression of RNA level by combining with the target gene, thereby influencing related metabolic pathways and biosynthesis processes [53]. In addition, these miRNAs are enriched in pathways related to cancer, such as colorectal cancer and the p53 signaling pathway. We hypothesized that these cancer-related genes may cause the significant miRNA variations observed in the 48–52 age group. This is similar to the increased incidence of colorectal cancer and other related cancers typically discovered in individuals aged 50–60, with incidence increasing with age [[54], [55], [56]]. There are different miRNA expression patterns in the plasma or serum of patients with colorectal cancer during the occurrence and development. These circulating miRNAs can be used as non-invasive biomarkers for the diagnosis and prognosis of colorectal cancer [[57], [58], [59]]. The p53 gene is widely recognized in scientific research for its anticancer effects and is considered one of the most relevant genes in human tumors. P53 acts as a "guardian" of the genome by regulating key biological processes such as the cell cycle, apoptosis, and DNA repair, effectively halting cancer development [60]. Furthermore, p53 finely balances a healthy lifespan, tumor suppression, and accelerated aging by regulating various mechanisms of the DNA damage response (DDR) [61]. P53 plays a crucial role in maintaining genome stability, and its mediation of ferroptosis is closely linked to aging-related features [62]. Numerous studies have confirmed the role of p53 in the aging process and age-related disorders [[63], [64], [65], [66], [67]]. Therefore, we believed that these cancer-related pathways may be important contributors to miRNA variation in this age group, and we may need to take into account the influence of these disease-associated miRNAs in later studies.

To identify additional age-related markers, we screened for age-related miRNAs using the lasso regression method. Lasso regression is an effective feature selection method that has been widely applied in previous research on age-associated molecular markers, demonstrating its reliability in the field of age prediction [4,68]. We further quantified the feature importance of preliminary models using the SHAP algorithm. Compared to traditional feature importance measures, SHAP analysis provide more intuitive and interpretable assessments of feature importance and are more robust to feature interactions [69]. Through lasso regression and SHAP analysis, we selected 38 miRNAs that are most predictive of age. Among these, the two miRNAs with the highest SHAP values in the optimal model were miR-500a-5p and miR-141-3p. Existing studies have shown that miR-141-3p is an important molecular marker related to aging and is involved in the regulation of growth hormone [70]. As age increase, the expression level of miR-141-3p in tissues and organs rises, and inhibiting its expression can improve skeletal muscle health [71]. BAP1, the target gene of miR-141-3p, inhibits PI3K activity, thereby inhibiting cell growth in Drosophila and humans [72]. In addition, the expression of miR-141-3p is downregulated in the elderly patients with Parkinson's disease, a neurodegenerative disease with high incidence [73]. These findings suggest that miR-141-3p is likely one of the key miRNAs controlling cellular aging. On the other hand, miR-500a-5p is primarily reported in cancer-related studies, such as those on colorectal cancer, melanoma and breast cancer [[74], [75], [76]]. The enrichment pathways of differentially expressed miRNAs in the 48–57 age group are also associated with these cancers, indicating that this age group may be an important period to study key miRNA changes throughout the life span. In the selected 38 age-related miRNAs, two members of the miR-29 family were identified. The miR-29 family is closely associated with the regulation of collagen, and the ability to synthesize collagen declines with age, leading to signs of aging [77]. Therefore, there is a significant correlation between the miR-29 family and age. Research indicates that miR-29 can regulate cardiac aging [78] and skeletal senescence [79], and its reduction may extend lifespan in progeria model [80].

To validate our screening of age-related miRNAs, we further used machine learning to construct age prediction models. The results showed that the elastic net model had the best performance. This may be because the elastic net, with its linear combination of L1 and L2 regularization, can effectively handle high-dimensional data and mitigate the risk of overfitting, which is consistent with our findings. The other three models performed poorly, likely because the selected final features were derived from lasso regression. Decision tree-based ensemble learning algorithm and neural network may not be suitable for capturing the complex relationship between miRNA screened based on lasso regression method and age. Notably, the neural network model suffered from overfitting, which may be related to the limited training data [81]. Finally, we constructed an elastic net model consisting of 38 miRNAs, which yielded a MAE of 4.09 years in age prediction. This accuracy is comparable to that of other types of molecular markers reported [82], indicating that blood miRNA has good application prospects in age prediction. Compared with the results of previous studies using miRNA-based age prediction model [26], our results show significant improvement. However, there is still a notable gap compared to the results obtained by integrating miRNA and piRNA (MAE = 3.171 years) [83], and the number of miRNA markers selected in their study is also relatively small. Therefore, in future study, we can consider further reducing the number of age-related markers and jointly applying other markers to optimize our machine learning model and improve performance.

Previous studies have found that sex can influence the association patterns between biomarkers and age [84]. To further investigate this, we performed model validation separately for male and female samples. Our results found less error in the male sample compared to the female sample, consistent with prior research [11,68]. This difference may stem from inherent differences between men and women in factors such as genetic background, hormone levels, and lifestyle, which may lead to different molecular patterns in their aging processes [85]. In our study, the elastic net model based on male samples has a MAE of 4.14 years, while for female samples, the MAE is 6.61 years. Despite the performance on female samples being inferior to that on male samples, the difference in errors (2.47 years) is not significant. Furthermore, even though the error in the female model is relatively high, it shows a smaller gap compared to other machine learning models based on miRNA [26]. This indicates that the miRNA selected in our study and the elastic net model is less influenced by sex, showing broad potential for future practical applications.

Although our study showed smaller errors compared to existing miRNA-based age prediction models, research on miRNA markers in the field of age prediction is still in its initial stages, and this study has certain limitations. Firstly, our study only used RNA-Seq data from blood samples for feature selection and model construction and has not been validated on new datasets. Additionally, the sample size is relatively small, which may affect the accuracy of the results. In the next step, we need to conduct large-scale validation using multiple types of samples and validate the application in practice. Secondly, the number of selected markers (38 miRNAs), may be too high and relatively difficult to apply to investigative clues in forensic scenarios. Future studies are needed to further screen the number of markers to meet the needs of practical applications. Thirdly, previous studies have combined miRNA markers with other biomarkers to construct multi-omics age prediction models, thereby enhancing prediction accuracy [83,86,87]. Therefore, in future research, we can consider incorporating more biomarkers to enhance predictive accuracy. In particular, lncRNA, which has gained significant attention in recent years and has been shown to have a relationship with age [88] and aging [89], may serve as effective markers for age estimation. Despite these limitations, this study has provided a solid foundation for using miRNA as a biomarker for age estimation.

## 5 Conclusion

In summary, this study utilized machine learning approaches to identify age-associated biomarkers from blood miRNA expression profiles and constructed corresponding predictive models. Through lasso regression analysis and SHAP analysis, we screened a set of 38 miRNAs with the highest age predictive capacity. Among the five machine learning models evaluated, the elastic net model and support vector machine model exhibited the best predictive performance, achieving MAE of 4.08 years and 4.19 years, respectively. This study identified age-related miRNA markers and the optimized modeling strategies established an important foundation for further research in this field. In future work, expanding the sample size, integrating multi-omics data, and refining the modeling details hold promise to further improve the performance and practical utility of miRNA markers in age prediction.

## CRediT authorship contribution statement

**Yanfang Lu:** Writing – original draft, Validation, Software, Methodology, Formal analysis, Data curation. **Anqi Chen:** Writing – review & editing, Methodology, Formal analysis, Data curation. **Mengxiao Liao:** Software, Methodology. **Ruiyang Tao:** Investigation. **Shubo Wen:** Investigation. **Suhua Zhang:** Writing – review & editing, Supervision, Resources, Project administration, Funding acquisition, Conceptualization. **Chengtao Li:** Supervision, Resources, Project administration, Funding acquisition, Conceptualization.

## Informed consent statement

Informed consent was obtained from all subjects involved in the study.

## Ethical Approval

The study was conducted in accordance with the Declaration of Helsinki, and approved by the Ethics Committee of the Academy of Forensic Science. Ministry of Justice, P.R. China.

## Funding

This work was supported by grants from the project supported by the Major Program of the 10.13039/501100001809National Natural Science Foundation of China (No. 82293650, No. 82293654).

## Declaration of competing interest

The authors declare that they have no known competing financial interests or personal relationships that could have appeared to influence the work reported in this paper.
